# Ecological phage therapy: Can bacteriophages help rapidly restore the soil microbiome?

**DOI:** 10.1002/ece3.70185

**Published:** 2024-08-13

**Authors:** Tarryn Davies, Christian Cando‐Dumancela, Craig Liddicoat, Romy Dresken, Rudolf H. Damen, Robert A. Edwards, Sunita A. Ramesh, Martin F. Breed

**Affiliations:** ^1^ College of Science and Engineering Flinders University Bedford Park South Australia Australia; ^2^ School of Biological Sciences and the Environment Institute University of Adelaide Adelaide South Australia Australia; ^3^ HAN University of Applied Sciences Nijmegen Netherlands

**Keywords:** bacteriophage, inoculation, microbiome, restoration genomics

## Abstract

Soil microbiota underpin ecosystem functionality yet are rarely targeted during ecosystem restoration. Soil microbiota recovery following native plant revegetation can take years to decades, while the effectiveness of soil inoculation treatments on microbiomes remains poorly explored. Therefore, innovative restoration treatments that target soil microbiota represent an opportunity to accelerate restoration outcomes. Here, we introduce the concept of ecological phage therapy—the application of phage for the targeted reduction of the most abundant and dominant bacterial taxa present in degraded ecosystems. We propose that naturally occurring bacteriophages—viruses that infect bacteria—could help rapidly shift soil microbiota towards target communities. Bacteriophages sculpt the microbiome by lysis of specific bacteria, and if followed by the addition of reference soil microbiota, such treatments could facilitate rapid reshaping of soil microbiota. Here, we experimentally tested this concept in a pilot study. We collected five replicate pre‐treatment degraded soil samples, then three replicate soil samples 48 hours after phage, bacteria, and control treatments. Bacterial 16S rDNA sequencing showed that phage‐treated soils had reduced bacterial diversity; however, when we combined ecological phage therapy with reference soil inoculation, we did not see a shift in soil bacterial community composition from degraded soil towards a reference‐like community. Our pilot study provides early evidence that ecological phage therapy could help accelerate the reshaping of soil microbiota with the ultimate aim of reducing timeframes for ecosystem recovery. We recommend the next steps for ecological phage therapy be (a) developing appropriate risk assessment and management frameworks, and (b) focussing research effort on its practical application to maximise its accessibility to restoration practitioners.

## Graphical abstract

In this study, we introduce the new concept of ecological phage therapy—the application of bacteriophages (viruses that infect bacteria) for the targeted reduction of the most abundant and dominant bacterial taxa present in degraded ecosystems, such as soil ecosystems. We ran a pilot study to test ecological phage therapy on soil and used 16S rDNA amplicon sequencing to assess changes in bacterial communities post phage and/or bacteria‐treated soils. We show a reduction in bacterial alpha diversity in phage‐treated soils, while soil bacterial community composition was largely unchanged in bacteria‐treated soil.

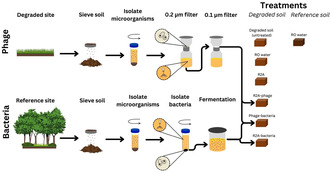

## 1 INTRODUCTION

Ecosystem degradation is a significant global issue that affects both ecosystem processes and biodiversity. Consequently, there have been repeated calls to scale up restoration interventions and to innovate new restoration practices (Mohr et al., 2021). Common restoration practices include the removal of invasive species and/or re‐introducing native plant communities, with the hope that these actions will initiate a broader recovery of the ecosystem (Benayas et al., 2009). However, some ecosystem components can take years to decades to recover post‐treatment. One critical ecosystem component that is rarely directly targeted and can be slow to restore is soil microbiota (Watson et al., 2022)—the community of microbes (e.g. bacteria, fungi, viruses) in a given environment.

Soil microbiota are essential for many ecosystem processes, including a range of biogeochemical processes (e.g. nutrient cycling, soil formation) and many plant symbiotic relationships (Bulgarelli et al., 2013; Fierer, 2017). Across many ecosystems and various types of ecosystem degradation (e.g. mining, agriculture, exotic plant invasions), soil microbiota from degraded ecosystems generally differ from remnant ecosystems in the composition of both taxa and functional potential (Liddicoat et al., 2019; Louisson et al., 2023; Watson et al., 2022). These altered soil microbial communities, along with changes in soil chemistry and plant composition in degraded ecosystems, can present barriers to restoration by altering plant–soil–microbial interactions and feedbacks (e.g. recovery of native vegetation; Suding et al., 2013; Pickett et al., 2019). As such, they should be considered when establishing restoration targets (Breed et al., 2019). In a restoration context, it can be difficult to discern whether soil microbiota represent drivers of ecosystem recovery (e.g. supporting plant communities) or whether they are followers of macro‐ecosystem changes (Harris, 2009).

Ecosystem restoration approaches need to be informed by reference ecosystems—that is, ecosystems that represent a benchmark for a restoration project (Gann et al., 2019). These reference ecosystems are usually near the restoration project site and contain the community of organisms (including soil microbiota) that represent the desired ecological state of restoration efforts. Restoration approaches, such as native plant revegetation, can shift soil microbiota compositions towards a restored state after eight or more years (Watson et al., 2022). Given that soil microbiota appear to take many years to respond to traditional restoration actions and may be proximal drivers to broader ecosystem restoration (Harris, 2009), innovative approaches that target soil microbiota are needed (Farrell et al., 2020; Yang et al., 2022). Here, we propose a new approach—ecological phage therapy—that could help facilitate rapid restoration of microbiota in target habitats such as soil. We define ecological phage therapy as the application of phages for the targeted reduction of the most abundant and dominant bacterial taxa present in degraded ecosystems. This targeted reduction represents an interim step towards recovery of a reference‐like state as it may reduce the abundance of resident bacterial taxa that present barriers to restoration (e.g. due to altered plant–soil–microbial feedbacks) and could potentially enhance the success of soil inoculation via decreasing bacterial diversity (i.e. low diversity soil bacterial communities are generally more receptive to establishment of introduced bacterial species than high diversity soils; Van Elsas et al., 2012).

Bacteriophages (referred to hereafter as ‘phages’) are viruses that specifically infect bacteria and are found in nearly every environment where their corresponding bacterial hosts are present. Phages are even more abundant than their bacterial hosts, outnumbering them 10:1 in most environments (Clokie et al., 2011). They are extremely host‐specific, and generally one phage will only infect a few strains of a particular bacterial species (Hatfull et al., 2022; Hyman & Abedon, 2010). Two common phage lifecycles are the lytic and lysogenic lifecycles. The lytic cycle involves the phage replicating inside a host bacterial cell and lysing the cell upon exiting. The lysogenic cycle involves the phage incorporating its DNA into the genome of its bacterial host.

Phage therapy uses lytic phages to target specific bacterial taxa to infect and kill them, resulting in control of the target bacterial populations (Sulakvelidze et al., 2001). Phage therapy was first investigated in medicine as an option for treating bacterial infections in humans (Roux, 1917; Twort, 1915). Unlike antibiotics, phage therapy should only kill the target pathogenic bacteria and theoretically should not impact non‐target bacterial strains (Sulakvelidze et al., 2001). It is also currently in development in agriculture as a means of biocontrol of antibiotic‐resistant pathogenic bacteria (Zhao et al., 2019) and bacterial crop pathogens, such as bacterial wilt disease (*Ralstonia solanacerum*; Wang et al. (2019)). In these instances, specific phages (one phage type, phage cocktails, or polyvalent phages) were isolated from and subsequently applied back to soil to reduce the abundance of target bacteria. While research has shown successful instances of phage therapy in soil environments, the commercial use in agricultural settings is still limited (Buttimer et al., 2017).

To the best of our knowledge, phage therapy has not been investigated in the context of ecosystem restoration. While ecological phage therapy uses the same principles as phage therapy in agriculture and medicine, we propose a key difference: Instead of targeting specific unwanted bacterial strains, we propose to apply phages for the targeted reduction of the most abundant and dominant bacterial taxa present in degraded soil, as these taxa are likely to be the most active and are potentially facilitating the maintenance of a bacterial community in a degraded state. This action is based on the ‘kill‐the‐winner’ principle of phage biology: the most abundant bacterial taxa in an environment are targeted and killed by phages from that environment (Thingstad, 2000; Weinbauer, 2004). These phage‐host dynamics lead to the expectation that high phage abundances in soil will be accompanied by proportional reductions in the abundances of their bacterial hosts—an effect that will be most pronounced in the more abundant taxa.

We propose that through ex situ repeated increases to the concentration of phages from degraded soil, and subsequently applying this high concentration phage medium to degraded soil destined for restoration, the abundance of the most dominant bacterial taxa—that is, those characteristic of a degraded bacterial community—would decrease. If this phage‐treated degraded soil is then inoculated with a bacterial community isolated from an ecological reference site, then a rapid shift and establishment of a restored soil microbiota may be possible.

Here, we ran an exploratory experimental study of ecological phage therapy in a laboratory. We applied treatments that used varying combinations of degraded soil phages, reference soil bacteria, and growth medium to soil sourced from a degraded ecosystem. Phages were isolated from the same source as the soil medium used in this study as the effect of this isolated community would likely be greater than that of a phage community isolated from a different soil source given the constant co‐evolution of phage and bacterial communities (Koskella & Brockhurst, 2014). We used high‐throughput amplicon sequencing of the bacterial 16S rRNA gene to characterise changes to the bacterial community before and 48 hours after treatments. Using this experimental framework, we addressed the following questions: (1) Can phages from degraded soil be used to reduce the diversity of bacteria from degraded soil? (2) Can phages from degraded soil be used to reduce the relative abundance of the most dominant bacteria from degraded soil? (3) Can subsequent addition of reference soil bacteria post‐phage treatment result in a shift in the bacterial community towards a restored community?

## 2 METHODS

### 2.1 Soil sample collection

Soils were collected from degraded and reference ecosystems at Mt Bold Reserve, South Australia (35.13° S, 138.68° E). These are the same sites used in our earlier work, and we provide a detailed description of their history and ecology in Liddicoat et al. (2020). Briefly, the degraded site was cleared and replaced by a grassland dominated by introduced grasses >100 years ago for small livestock grazing until 2003, when South Australia's water utility took over management of this site. It has been mowed annually to form a firebreak since 2003. The reference ecosystem is a mostly intact grassy woodland and provides a target ecosystem for regional restoration efforts. The close geographic proximity of the degraded and reference ecosystems provides a good sampling site for this study as the soil chemistry is similar across the area with the main difference being the state of degradation of the ecosystem (see Table S1 for soil physicochemical data from Liddicoat et al., 2020).

**FIGURE 1:**
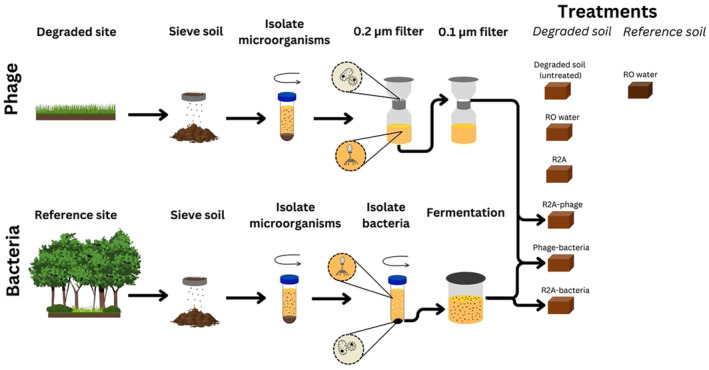
Schematic of our experimental design, showing the steps involved in phage (top) and bacteria (bottom) concentration pathways, plus the four different experiment treatments and controls.

In September 2019, we collected and pooled the top 10 cm of soil from nine sites within a 3 × 3 m plot at both the reference and degraded sites (Figure 1). The day after collection, the soil was sieved using a 25 mm sieve to remove roots, rocks, and non‐soil particles, creating a homogenised soil mix. The soil was stored at 4°C before and after sieving until microbial suspensions were made 5 days later (described below).

### 2.2 Soil microbe R2A suspension

A soil bacteria and phage suspension were isolated from reference and degraded soil, separately. This involved suspending soil in R2A medium (R2A preparation outlined in Supporting Information), centrifuging at 454 *g* for 5 min and collecting the microbe‐laden R2A supernatant. We obtained 1500 mL of microbe‐laden R2A from 500 g of each of the soils.

### 2.3 Phage isolation

The degraded soil supernatant was then filtered using a 0.2 μm filter to separate bacteria from phages. The solution was filtered a second time to increase the concentration of phages by using a 0.1 μm filter. The phages in the 0.1 μm filter were then resuspended in 50 mL of R2A medium—the ‘phage solution’.

### 2.4 Bacteria isolation

The reference soil supernatant was centrifuged at 4779 *g* for 15 min to create bacterial pellets. The pellets were resuspended in 15 mL R2A medium. This resuspension was then added to 285 mL R2A medium (1:20 ratio), covered and left to anaerobically ferment at room temperature for 24 h to increase the concentration of bacteria in the solution. After fermentation, the R2A‐bacteria solution was divided into 50 mL tubes and centrifuged at 5000 rpm for 15 min; pellets were pooled and resuspended in 50 mL by serial re‐suspension—the ‘reference bacteria solution’.

### 2.5 Experimental setup

We had five experimental treatments, each added to degraded soil, as follows: (1) 6 mL reverse‐osmosis (RO) water (Control); (2) 6 mL R2A medium only; (3) 3 mL R2A and 3 mL phage solution; (4) 3 mL R2A and 3 mL reference bacteria solution; (5) 3 mL phage solution and 3 mL of reference bacteria solution. Our sixth treatment comprised reference soil with 6 mL of RO water added. Each treatment was replicated three times, each in a 9 × 9 × 4 cm sterile plastic box, giving a total of 18 samples (15 with degraded soil, 3 with reference soil). Each box contained 250 g of soil and was covered by an air conditioner filter to minimise contamination but maintain aerobic conditions. Boxes were randomly allocated a position within a constant temperature room at 25°C. Soils were stirred at 1, 8, 24, and 48 h with sterilised spoons.

### 2.6 Data collection

After 48 h, soil was stirred in each replicate and 2 mL of soil was collected. Soil samples were stored at −20°C until DNA extraction. In addition, we collected five untreated degraded soil samples that did not go through the 48‐h 25°C incubation process. This gave our study a total of 23 soil samples (DNA extraction, 16S rRNA gene PCR‐amplification, DNA sequencing, and construction and filtering of amplicon sequencing variants (ASVs) methods are described in Supporting Information).

### 2.7 Statistics

We used R (V4.2.1) for all statistics (R Core Team, 2021), using *Phyloseq* (McMurdie & Holmes, 2013) to manage the bacterial community abundance datasets. Data cleaning and decontamination steps are detailed in the supplementary methods. Following data cleaning, samples were rarefied to the sample with the lowest read depth (*n* = 28,545 reads from an R2A‐Phage sample). Rarefied samples were then used to estimate the alpha diversity in the samples by determining the effective number of ASVs (calculated as exp(Shannon's diversity index); Jost (2006)). We used general linear models (LMs) to test the effect of treatment on the effective number of ASVs at 48 h. Model residuals were assessed for normality with Shapiro–Wilk tests and homogeneity of variance was assessed with Levene's tests. Beta diversity among samples was visualised using non‐metric multidimensional scaling (NMDS) of Bray‐Curtis distances of rarefied ASV abundances. We used PERMANOVAs with the *adonis* test in *Vegan* (Oksanen et al., 2020) to examine the effect of treatment (i.e. testing for differences in the centroids of NMDS coordinates to indicate differences in composition between treatments). We used *permutest* in *Vegan* (Oksanen et al., 2020) to test for homogeneity of multivariate dispersions.

Prior to differential abundance analysis, we excluded reference and untreated degraded soil samples and then removed ASVs that did not appear in two or more samples. We then used the glm.aldex function in ALDEX2 (ANOVA‐Like Differential Expression tool for high throughput sequencing data) for differential abundance analysis (Fernandes et al., 2013, 2014; Gloor et al., 2016). ALDEX2 uses Monte‐Carlo instances sampled from the Dirichlet distribution to determine the technical and statistical error. The generalised linear model function of ALDEX2 was used to measure the differential abundance of ASVs between the degraded soil + RO water treatment (Control) and all other treatments (excluding reference and untreated degraded soil). We did not include untreated degraded soil as these samples were taken at a different time point to all other samples (i.e. before treatments were applied). We were only interested in changes in the abundance of the most dominant ASVs in the degraded soil + RO water Control, as such we only looked at the ALDEX2 output for the top 200 most abundant ASVs in the Control treatment. We defined the top 200 most abundant ASVs as the ASVs with the highest median of the centred log‐ratio (CLR)‐transformed Monte‐Carlo instances for the Control treatment group. Following on, we adjusted *p*‐values using the Benjamini‐Hochberg method to reduce the false discovery rate.

## 3 RESULTS

A total of 1,495,520 bacterial 16S rRNA reads were obtained from the 23 soil samples, after filtering. The number of reads per sample ranged from 28,545 to 128,391, and the mean number of reads was lowest for reference soils (summary statistics for read counts and ASV alpha diversity are provided in Table S2).

**FIGURE 2:**
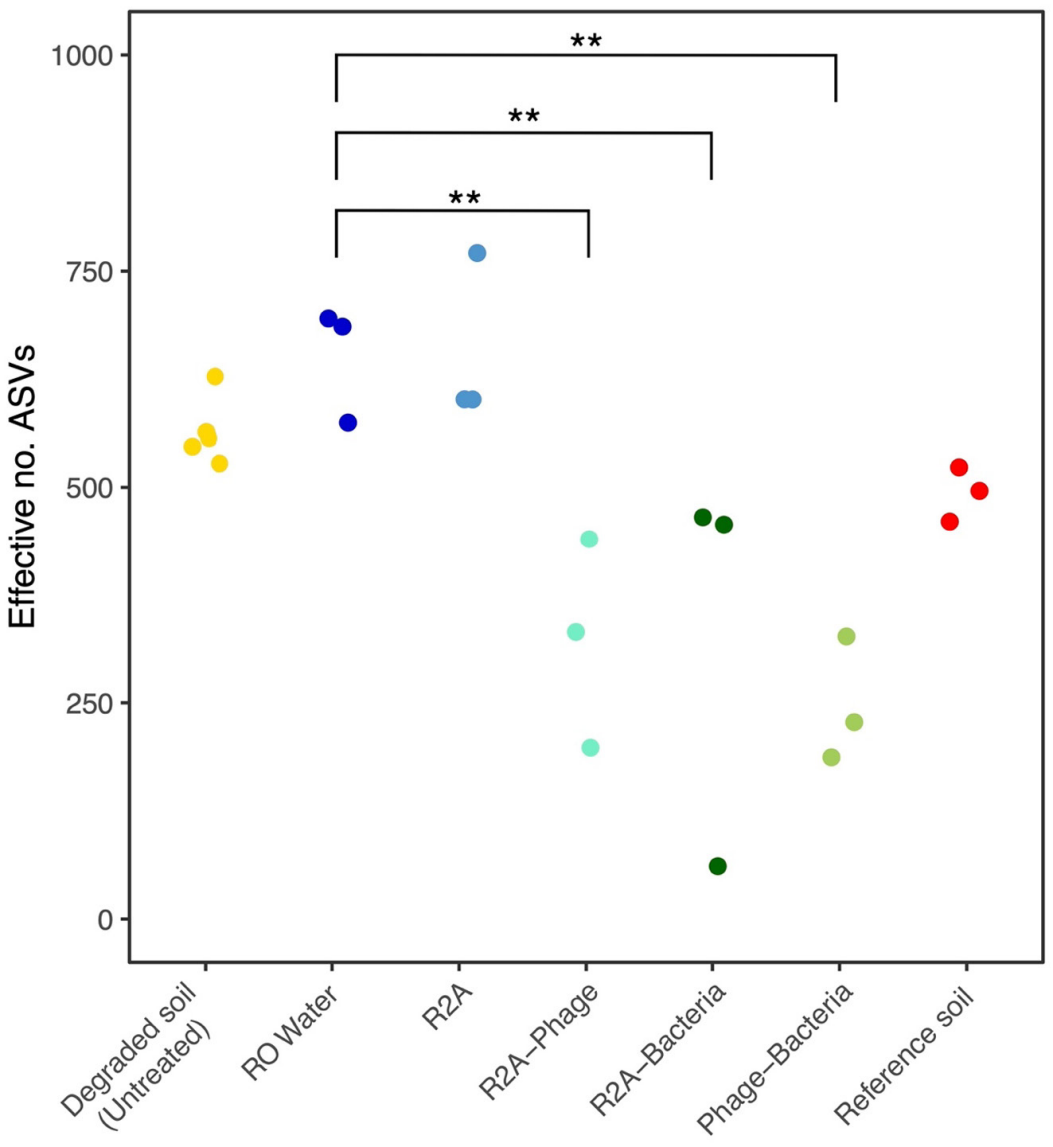
Effective number of ASVs (i.e. exp (Shannon diversity index)) for the initial untreated degraded soil, the Control (degraded soil + RO water), the four experimental treatments, and reference soils at 48 hrs, based on rarefied ASV abundance data (28,545 reads). Pairwise tests were used to determine significant differences in the effective number of species in treatments compared to the control (degraded soil + RO Water). **p < .01.

Treatment had a significant effect on the effective number of ASVs (LM: df = 5 and 12, *F* = 6.46, *p* = .004, Figure 2). There were significantly lower effective number of ASVs in the treatments that included the addition of bacteria and/or phage compared to the water control. The phage‐bacteria treatment had a significantly lower effective number of ASVs compared to the degraded soil + RO water Control treatment (phage‐bacteria = 247 vs. Control = 652 effective number of ASVs; *p* = .002). The R2A‐phage treatment also had a significantly lower effective number of ASVs compared to the Control treatment (R2A‐phage = 323 ASVs; *p* = .006). Similarly, the R2A‐bacteria treatment had a mean effective number of ASVs of 328 which also was significantly different to the Control (*p* = .006). The effective number of ASVs in the R2A treatment and reference soil did not significantly differ from the Control treatment (*p* > .05).

**FIGURE 3:**
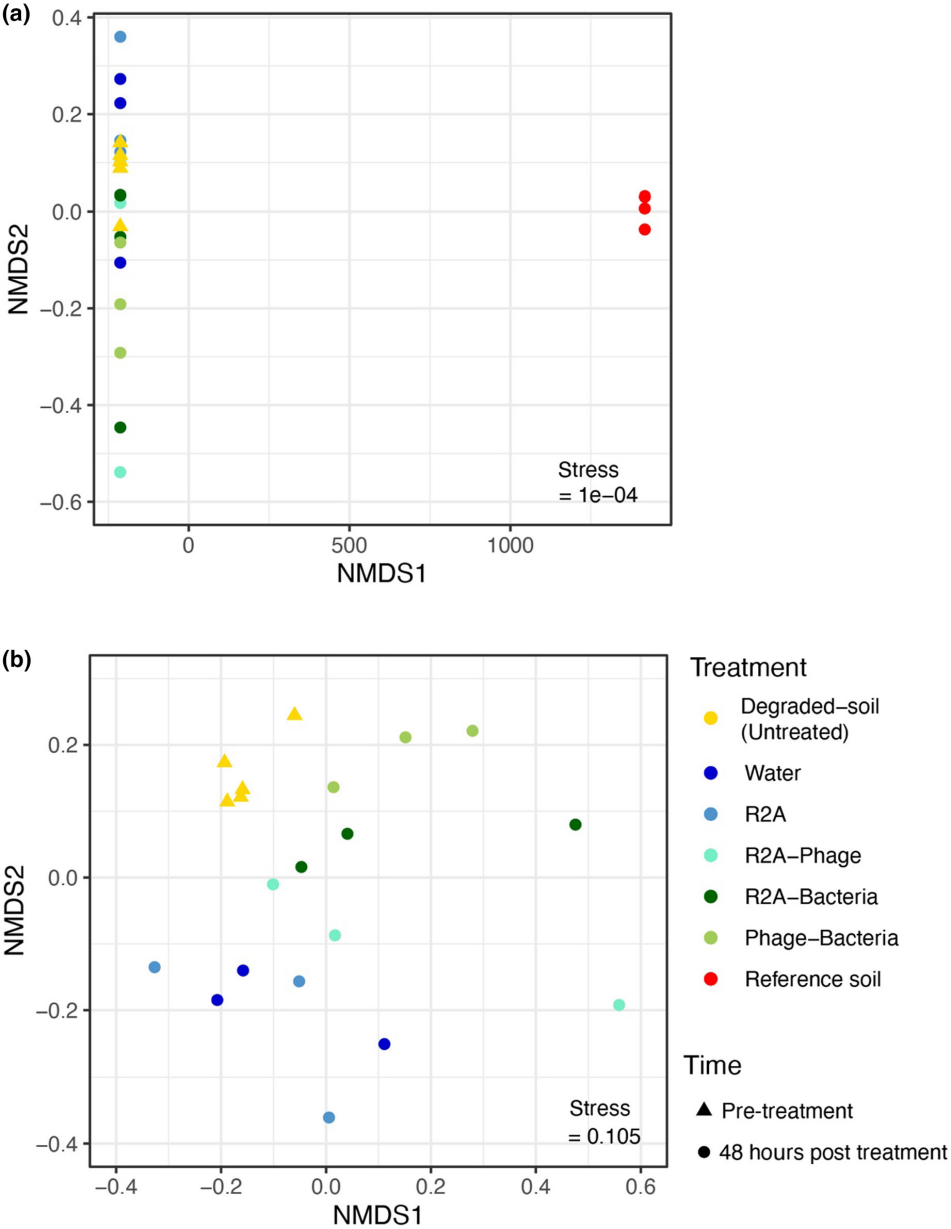
Ordination plots using non‐metric multidimensional scaling (NMDS) and Bray Curtis dissimilarity for all treatments (a) and excluding the reference soil (b), based on rarefied ASV abundance data (28,545 reads). Each point represents the bacterial community within a sample and closer points have more similar community compositions.

Bacterial community composition differed significantly by soil treatment/source (including untreated reference and degraded soil), which explained 81% of variation in composition (PERMANOVA: df = 6, permutations = 999, *F* = 11.39, *p =* .001; Figure 3a). Reference soil bacterial community compositions were distinct from all other soil treatment groups (Figure 3a). As such, we performed another PERMANOVA with reference soil excluded to look at differences within the treatments performed on degraded soils. Degraded (untreated) soil was also excluded from this second PERMANOVA test as this sample did not go through the 48‐h, 25°C incubation process, and we did not expect it would provide a fair comparison to the other incubated/treated samples. This second PERMANOVA result showed that treatment had a significant effect on bacterial community composition, with treatment explaining 65% of variation in composition (PERMANOVA: df = 4, permutations = 999, *F* = 4.65, *p* = .001, Figure 3b).

**FIGURE 4:**
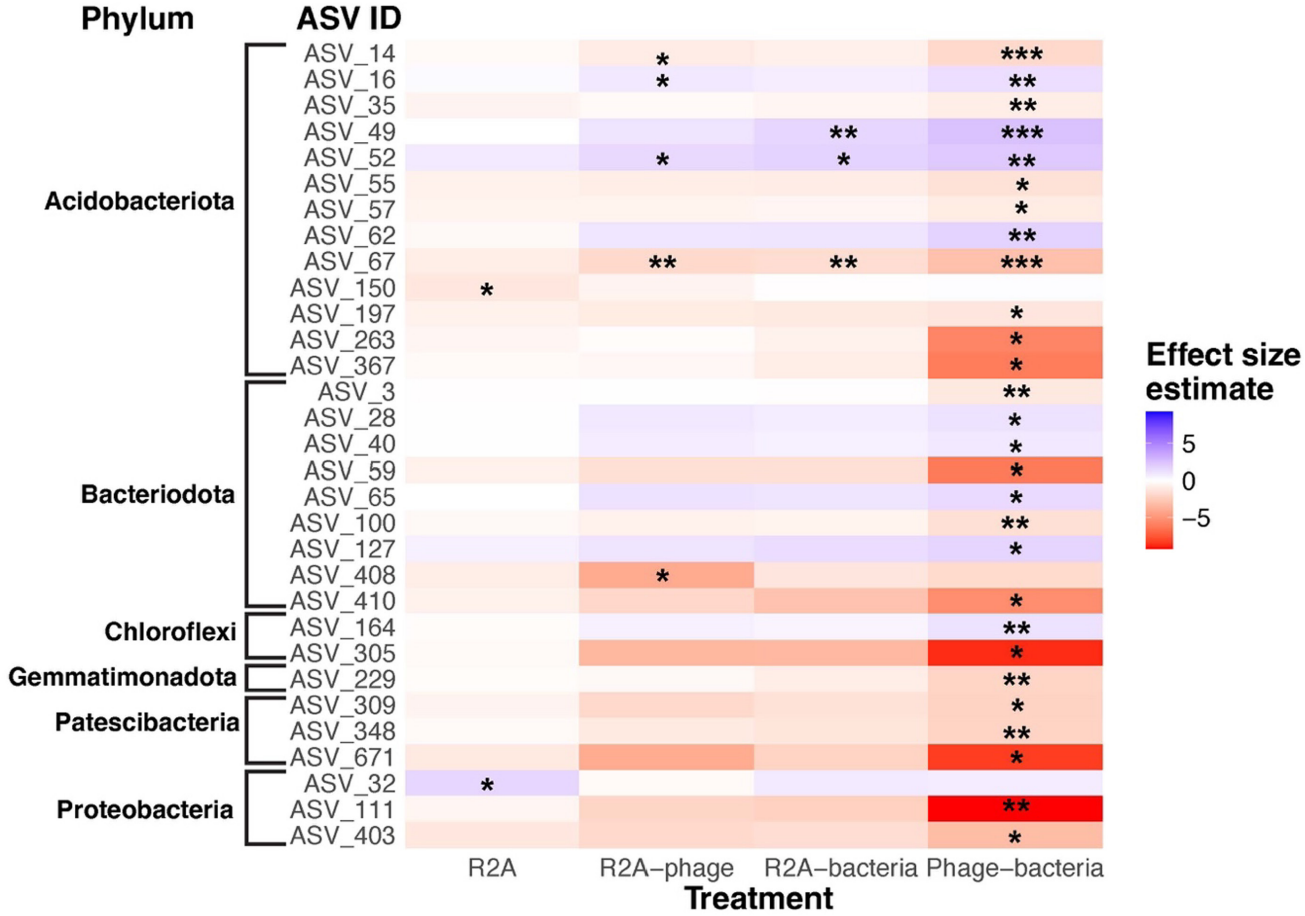
Heatmap of ASVs found to be significantly different from the Control (degraded soil + RO water) using ALDEX2. *p < .05, **p < .01, ***p < .001. The effect size estimate units portrayed in the heatmap are coefficients of a generalised linear model. Full taxonomic descriptions can be found in Table S3.

There were 31 ASVs out of the 200 most abundant ASVs in the Control that were significantly different in at least one of the treatments (Figure 4). Twenty‐eight out of the 31 significantly different ASV abundances were found in the phage‐bacteria treatment, of which 19 of these ASVs had lower abundance and 9 had higher abundance in the phage‐bacteria treatment (Figure 4). There were five differentially abundant ASVs in the R2A‐phage treatment, three had lower and two had higher abundance in the R2A‐phage treatment (Figure 4). Only three ASVs were differentially abundant in the R2A‐bacteria treatment, two of which were more abundant in the R2A‐bacteria treatment. Two ASVs were differentially abundant in the R2A treatment compared to the Control, with one less and one more abundant in the R2A treatment (Figure 4). Taxonomic descriptions for the significantly different ASVs in the treatments compared to the Control treatment (RO water) are reported in Table S3.

## 4 DISCUSSION

Our exploratory study tested the potential for ecological phage therapy to help accelerate the recovery of soil microbiota in an ecosystem restoration context. We found promising preliminary evidence that phage therapy could be used to assist in the rapid reshaping of soil microbiota. Specifically, we showed that treatments with phages (R2A‐phage and phage‐bacteria) generally had reduced soil bacterial diversity and collectively had a reduced abundance of ~10% of the most common bacterial taxa in the Control treatment (degraded soil + RO water). These findings are consistent with the action of an increased concentration of phages in the soil environment specifically targeting bacteria that are dominant in degraded soil. However, when we combined ecological phage therapy with an inoculation derived from reference soil, we were unable to demonstrate a shift in the soil bacterial community composition from degraded soil towards a reference‐like community.

We show that phage‐treated soil had reduced bacterial diversity, which is consistent with the ‘kill‐the‐winner’ principle and suggests that these treatments had increased abundances of phages. However, we did not directly quantify the phage abundances, and future studies should extend our study by further investigating such components of ecological phage therapy. While previous studies have successfully used phages to control specific bacteria pathogens, such as *Ralstonia solanacearum* (Wang et al., 2019), most of these studies target single species, instead of whole communities as we did here. Currently, little is known about the complex phage‐bacteria dynamics in soil, and even less on the impacts that altering entire phage communities has on bacteria community composition (Braga et al., 2020). There is some evidence that introducing phages into soils, whether during or after bacterial colonisation and under various soil conditions, does not consistently affect the alpha diversity or beta diversity of soil bacteria (Braga et al., 2020). The ‘kill‐the‐winner’ principle suggests that the most abundant bacterial taxa will be targeted and killed by phages from that environment. We showed that 19 out of the 200 most common bacterial taxa in the Control treatment (degraded soil + RO water) were significantly less abundant in the phage‐bacteria treatment. However, this extent of reduction in ASV abundance was not observed in the R2A‐phage treatment. We acknowledge that greater replication would provide stronger evidence; however, we were unable to run a power analysis to inform sample size as no pilot data was available.

We show that soil treatment had a significant effect on bacteria community composition. However, we failed to show a shift in the degraded soil bacteria community towards a reference‐like composition with the addition of bacteria from reference soil, even when applied with a phage treatment. Previous work has suggested soil inoculation can be unsuccessful (Emsens et al., 2022) or, in one case, weakened when inoculating topsoil (as used in this pilot study) as opposed to subsoil (Wubs et al., 2016). One possible explanation for unsuccessful inoculations is that the soil abiotic and microbial conditions are not conducive for inocula to establish. We did not reanalyse soil abiotic conditions in this study, but encourage future studies to collect contemporaneous soil data where directly comparable soil data are absent. Abiotic parameters, such as pH, can greatly influence soil bacterial community composition and can shift soils in different directions even when they receive identical inoculations (Yan et al., 2019). Introducing bacteria into a soil environment with an established bacteria community that is suited to the environment may result in the unsuccessful establishment of the introduced bacteria. Moreover, the bacterial community interactions (competition, facilitation) as well as ecological succession dynamics (priority effects, niche availability) all have a role in shaping soil bacterial assemblages (Debray et al., 2022) and could affect the inoculation success.

Another possible reason that the degraded bacterial soil assemblages did not shift towards a reference‐like community is the type of inoculant used. We used microbial suspensions for this study as it allowed for the separation of bacteria from phages in the soil. However, culturing soil bacterial communities in growth mediums may result in the cultured community not accurately representing the soil bacterial community (Steen et al., 2019). We did not characterise the suspension of reference soil bacteria or RO water and, as such, we are unable to confirm the extent to which the suspension of bacteria reflected the reference soil bacterial community or measure level of bacterial contaminants in the RO water. Further investigations into culturing media and soil inoculation are required to optimise these steps in ecological phage therapy.

**FIGURE 5:**
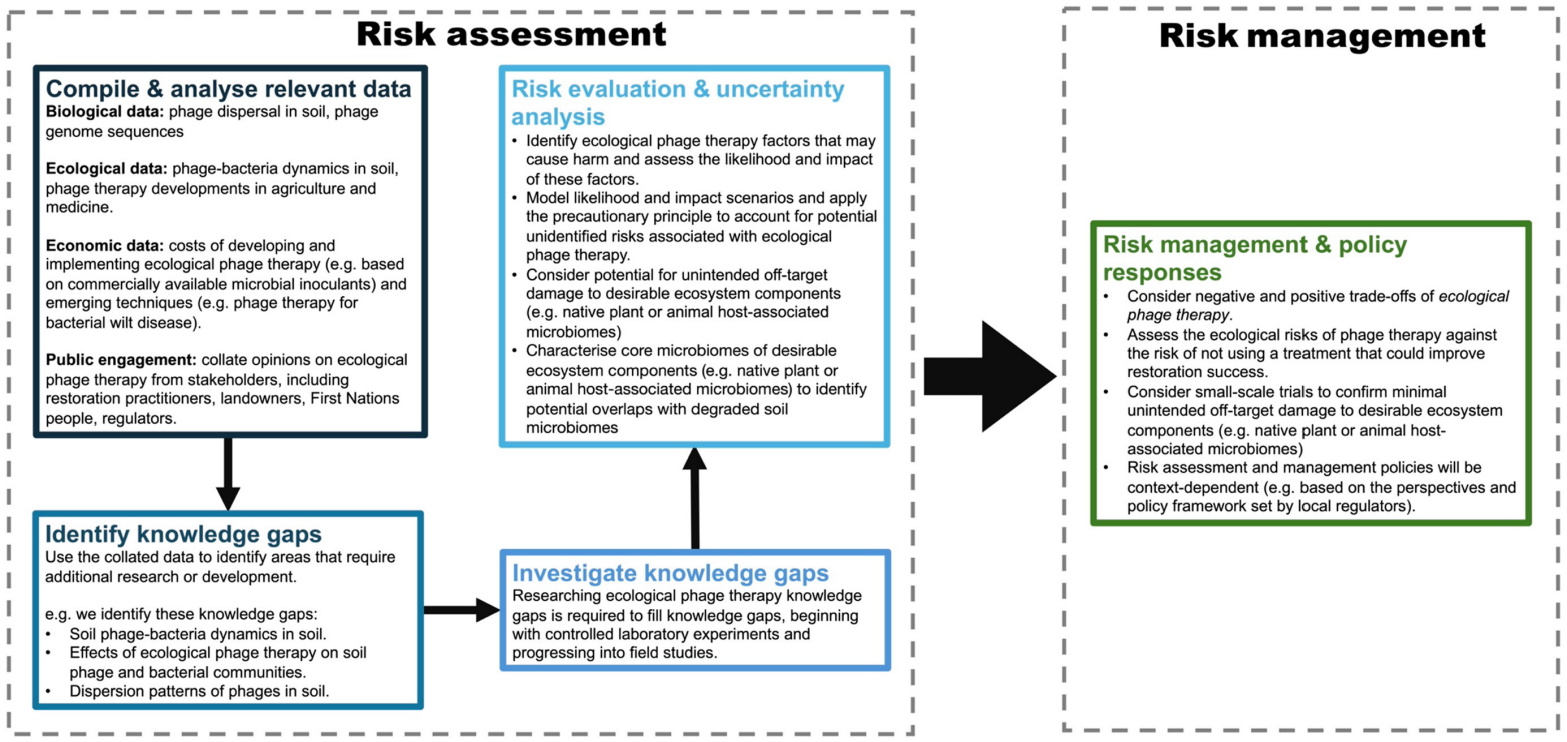
Example considerations for risk assessment and risk management of ecological phage therapy.

Our exploratory study also highlights that there is potential for ecological phage therapy to be incorporated into restoration practice. However, further research is needed to refine and improve this technique so that it is available to restoration practitioners. With continued refinement, there is potential to develop methods or products specifically tailored for use by restoration practitioners. Nonetheless, the required laboratory facilities and associated costs may limit the accessibility of ecological phage therapy for practitioners. We suggest future research directions include refining the methods used to isolate and concentrate both the bacterial inoculations from soils (as previously encouraged (Sáez‐Sandino et al., 2023); e.g. different artificial growth mediums) and the soil phage communities. In addition, we suggest that future studies are scaled up to increase statistical power (plus run power analyses to inform sample sizes). Detailed characterisation of the soil microbiota (e.g. using shotgun metagenomics) in treatments is needed to better understand which isolation approach is suitable plus to monitor the functional changes in microbial communities following treatments. Additionally, studies could include optimising the number of phages and bacteria applied to soils to achieve the desired community shift. These studies could include quantification steps, such as electron microscopy and flow cytometry for phages and optical density measurements for bacteria. Scaling up this research and extending it to field settings, plus developing an ecological phage therapy risk assessment and risk management frameworks to evaluate and manage unintended consequences (Breed et al., 2019), will be important next steps to progress ecological phage therapy (see Figure 5 for example risk assessment considerations). Further research is necessary to determine the practical feasibility of this technique and its effect on native vegetation establishment. The use of ecological phage therapy must weigh up the potential for restoration success against the cost and resources required.

## 5 CONCLUSIONS

Our study highlights the potential for ecological phage therapy to assist in rapidly re‐shaping soil microbiota which may have important applications in ecosystem restoration. We show that phage‐treated soils had less bacterial diversity compared to other treatments. However, in this exploratory work, we were unable to demonstrate our ultimate objective of shifting the phage‐treated degraded soil towards a reference‐like bacterial community, and additional research into ecological phage therapy is needed before it can be used in restoration practice.

## AUTHOR CONTRIBUTIONS

**Tarryn Davies:** Conceptualization (equal); data curation (equal); formal analysis (lead); investigation (equal); methodology (equal); writing – original draft (lead); writing – review and editing (equal). **Christian Cando‐Dumancela:** Data curation (equal); formal analysis (equal); investigation (equal); methodology (equal); project administration (equal). **Craig Liddicoat:** Formal analysis (equal); investigation (equal); methodology (equal); supervision (supporting); writing – review and editing (equal). **Romy Dresken:** Conceptualization (equal); writing – original draft (equal); writing – review and editing (equal). **Rudolf H. Damen:** Conceptualization (equal); data curation (equal); investigation (equal); methodology (equal); writing – original draft (equal); writing – review and editing (equal). **Robert A. Edwards:** Writing – review and editing (lead). **Sunita A. Ramesh:** Supervision (supporting); writing – review and editing (lead). **Martin F. Breed:** Conceptualization (equal); formal analysis (equal); investigation (equal); methodology (equal); project administration (equal); supervision (lead); writing – original draft (equal); writing – review and editing (equal).

## FUNDING INFORMATION

This research did not receive funding from any external funding bodies.

## CONFLICT OF INTEREST STATEMENT

The authors declare that there are no competing interests.

## Supporting information


Table S1
Table S2
Table S3

## Data Availability

Raw FASTQ files are available on the Sequence Read Archive (SRA) (http://www.ncbi.nlm.nih.gov/bioprject/1146961). Project data, metadata, and code are available on figshare (https://doi.org/10.6084/m9.figshare.25602615).
