# Supplementary material for: Ecological phage therapy: Can bacteriophages help rapidly restore the soil microbiome?
Source: Ecol Evol. 2024 Aug 13;14(8):e70185. doi: 10.1002/ece3.70185 (PMC11322231; doi:10.1002/ece3.70185)
Supplement: Supplementary file 1 — Table S1 Table S2 Table S3 [file ECE3-14-e70185-s001.pdf]

Supplementary information

Ecological phage therapy: can bacteriophages help rapidly restore the soil microbiome?

Running title: **Ecological phage therapy**

Tarryn Davies[1], Christian Cando-Dumancela[1], Craig Liddicoat[1], Romy Dresken[2], Rudolf H. Damen[1,3], Robert A. Edwards[1], Sunita A. Ramesh[1], Martin F. Breed[1]

[1]College of Science and Engineering, Flinders University, Bedford Park, SA 5042, Australia.

[2]School of Biological Sciences and the Environment Institute, University of Adelaide, SA 5005, Australia

[3]HAN University of Applied Sciences, Nijmegen, 6524 Netherlands.

Corresponding author: Martin Breed: College of Science and Engineering, Flinders University, Adelaide, SA 5001, Australia. telephone +61 8 8201 7877, email martin.breed@flinders.edu.au

Keywords: bacteriophage, inoculation, microbiome, restoration genomics

Supplementary methods

Preparation of the R2A medium

R2A medium (5x concentrated) was prepared by adding the following ingredients to ultra-pure water (milliQ):

- 2.5 g/L Casein acid hydrolysate
- 2.5 g/L Dextrin
- 1.5 g/L Di-potassium phosphate
- 0.12 g/L Magnesium sulphate
- 2.5 g/L Proteose peptone
- 1.5 g/L Sodium pyruvate
- 2.5 g/L Soluble starch
- 2.5 g/L Yeast extract

This solution was made to 1 L, which then had 4 L of ultra-pure water added to make a 5 L solution of R2A medium. This solution was stirred and homologized, after which it was adjusted to a pH of 7.2 (± 0.2 at 25°C) by citric acid/sodium hydroxide buffer (pH 5.5) with added fungicide.

DNA extraction and sequencing

DNA was extracted from all samples using the DNAeasy PowerLyzer Soil Kit, with the inclusion of an extraction blank control. In each sample, the 16S rDNA gene was PCR amplified using the forward 27F and reverse 519R primers, including the 12 bp Golay barcodes as described by (Caporaso et al., 2012) by the Australian Genome Research Facility (AGRF, Adelaide, Australia). 1U Immolase DNA polymerase (Bioline) was used per reaction with the following PCR protocol: 10 min activation at

95 °C, 35 cycles of 30 s at 94 °C, 10 s at 55 °C, and 45 s at 72 °C, and 10 min of final extension at 72 °C.

PCR products (ca. 530 bp) were visualised by electrophoresis in 2 % agarose gel 0.5 × TAE buffer. PCR plates showing bands for the negative controls were re-amplified until they appeared blank. Products were purified with Agencourt AMPure XP bead clean-up. Amplicon concentrations were quantified using the Quant-iT™ PicoGreen® dsDNA Assay Kit, normalised by diluting to 10nM with variable volumes of 10mM Tris (pH 8.5), and sized on an Agilent Bioanalyzer. Equal volumes of these normalised PCR products were then pooled and diluted to 4 nM, and then sequenced on an Illumina MiSEQ platform at AGRF.

Bioinformatics

Diversity profiling was completed using Qiime2.2022.2 (Bolyen et al., 2019). The Cutadapt plugin was used to remove primer sequences and quality filter the raw reads (Martin, 2011). Following this, forwards and reverse reads are combined and further filtered using DADA2 (Callahan et al., 2016). Amplicon sequence variants were determined from the SILVA version 138.1 rRNA database (Quast et al., 2013; Yilmaz et al., 2014; Glöckner et al., 2017) using the q2-feature-classifier (Bokulich et al., 2018) which utilises the classify-sklearn naïve Bayes taxonomy classifier.

16S rDNA data cleaning

We used R (V4.2.1) for all downstream statistical analysis (R Core Team, 2021), using *Phyloseq* (McMurdie & Holmes, 2013) to manage the bacterial community abundance datasets. ASVs were removed if they were not classified as bacteria at the phylum level, were taxa associated with mitochondria and chloroplasts, were

found in less than two samples or were assessed as contaminating sequences using the R decontam package with negative control data (Davis et al., 2018).

Table S1. Soil physicochemical properties from the degraded and reference site in August 2018, reported in (Liddicoat et al. 2020).

	**Reference site**			**Degraded site**		
**Sample no.**	1	2	3	1	2	3
Colour	Dark Gray	Dark Gray	Gray Brown	Gray Brown	Dark Gray	Dark Gray
Gravel (%)	0	5	5-10	5	0	
Clay (%)	8.17	23.39	11.91	10.23	7.85	7.16
Course Sand (%)	21.80	29.94	23.21	32.25	34.96	43.82
Fine Sand (%)	55.65	39.53	48.94	51.36	51.26	40.78
Sand (%)	77.45	69.47	72.15	83.61	86.22	84.60
Silt (%)	14.38	7.14	15.94	6.17	5.93	8.24
Moisture (%)	34.10	28.34	22.26	18.15	17.38	22.43
Conductivity (dS/m)	0.065	0.065	0.063	0.023	0.019	0.028
pH (H2O)	6.3	6.7	6.2	5.6	5.6	5.4
pH (CaCl2)	5.1	5.7	5.0	4.4	4.3	4.2
Organic Carbon (%)	3.13	2.82	2.86	1.92	1.56	1.81
Ammonium Nitrogen (mg/kg)	9	6	5	6	2	3
Nitrate Nitrogen (mg/kg)	3	6	6	2	2	4
Phosphorus Colwell (mg/kg)	8	5	5	12	11	10
Potassium Colwell (mg/kg)	124	185	146	38	21	38
Sulfur (mg/kg)	4.9	3.9	4.8	2.2	2.0	2.1
DTPA Copper (mg/kg)	2.03	0.56	0.44	0.18	1.35	0.17
DTPA Iron (mg/kg)	133.53	55.04	88.25	235.48	293.04	271.92
DTPA Manganese (mg/kg)	12.50	9.03	9.55	2.56	2.50	1.99
DTPA Zinc (mg/kg)	1.73	2.12	3.38	0.50	0.58	0.57
Exc. Aluminium (meq/100g)	0.043	0.045	0.085	0.743	0.542	0.516
Exc. Calcium (meq/100g)	7.89	10.55	6.20	1.44	1.31	1.39
Exc. Magnesium (meq/100g)	2.15	3.46	1.81	0.40	0.33	0.42
Exc. Potassium (meq/100g)	0.27	0.37	0.25	0.07	0.05	0.08
Exc. Sodium (meq/100g)	0.24	0.20	0.19	0.08	0.05	0.06
Boron Hot CaCl2 (mg/kg)	0.76	1.18	0.72	0.37	0.28	0.29

Table S2. Mean and standard deviation for 16S read count, ASV count and effective number of ASVs for each treatment.

Treatment	Mean read count ± SD	Mean ASV count ± SD (ASV richness)	Mean effective number of ASVs ± SD
Degraded soil (untreated)	68676 ± 14405	1541 ± 169	576 ± 40
RO water	60575 ± 11198	1530 ± 281	662 ± 74
R2A	75168 ± 46582	1687 ± 510	674 ± 114
R2A-phage	56826 ± 27282	1282 ± 463	330 ± 129
R2A-bacteria	73416 ± 7372	1379 ± 372	337 ± 238
Phage-bacteria	69089 ± 11907	1304 ± 230	250 ± 71
Reference soil	48973 ± 1955	1205 ± 35	500 ± 35

Table S3. Taxonomy for ASVs identified in treatments as having significantly different abundances compared to RO water using ALDEX2 CLR transformation.

ASV	Kingdom	Phylum	Class	Order	Family	Genus	Species
ASV_14	Bacteria	Acidobacteriota	Acidobacteriae	Bryobacterales	Bryobacteraceae	Bryobacter	Unclassified
ASV_16	Bacteria	Acidobacteriota	Acidobacteriae	Bryobacterales	Bryobacteraceae	Bryobacter	Unclassified
ASV_35	Bacteria	Acidobacteriota	Acidobacteriae	Acidobacteriales	Acidobacteriaceae_(Subgroup_1)	Occallatibacter	Unclassified
ASV_49	Bacteria	Acidobacteriota	Acidobacteriae	Acidobacteriales	Acidobacteriaceae_(Subgroup_1)	Terracidiphilus	uncultured_bacterium
ASV_52	Bacteria	Acidobacteriota	Acidobacteriae	Subgroup_2	Subgroup_2	Subgroup_2	uncultured_forest
ASV_55	Bacteria	Acidobacteriota	Acidobacteriae	Acidobacteriales	Koribacteraceae	Candidatus_Koribacter	uncultured_bacterium
ASV_57	Bacteria	Acidobacteriota	Acidobacteriae	Acidobacteriales	Acidobacteriaceae_(Subgroup_1)	uncultured	uncultured_Acidobacterium
ASV_62	Bacteria	Acidobacteriota	Acidobacteriae	Acidobacteriales	uncultured	uncultured	uncultured_bacterium
ASV_67	Bacteria	Acidobacteriota	Acidobacteriae	Subgroup_2	Subgroup_2	Subgroup_2	uncultured_forest
ASV_150	Bacteria	Acidobacteriota	Acidobacteriae	Acidobacteriales	Acidobacteriaceae_(Subgroup_1)	Granulicella	Unclassified
ASV_197	Bacteria	Acidobacteriota	Acidobacteriae	Subgroup_2	Subgroup_2	Subgroup_2	uncultured_bacterium
ASV_263	Bacteria	Acidobacteriota	Acidobacteriae	Solibacterales	Solibacteraceae	Candidatus_Solibacter	Unclassified
ASV_367	Bacteria	Acidobacteriota	Acidobacteriae	Acidobacteriales	Koribacteraceae	Candidatus_Koribacter	uncultured_bacterium
ASV_3	Bacteria	Bacteroidota	Bacteroidia	Sphingobacteriales	env.OPS_17	env.OPS_17	uncultured_Bacteroidetes
ASV_28	Bacteria	Bacteroidota	Bacteroidia	Sphingobacteriales	env.OPS_17	env.OPS_17	uncultured_bacterium
ASV_40	Bacteria	Bacteroidota	Bacteroidia	Sphingobacteriales	Sphingobacteriaceae	Mucilaginibacter	Unclassified
ASV_59	Bacteria	Bacteroidota	Bacteroidia	Sphingobacteriales	env.OPS_17	env.OPS_17	Unclassified
ASV_65	Bacteria	Bacteroidota	Bacteroidia	Sphingobacteriales	env.OPS_17	env.OPS_17	uncultured_Bacteroidetes
ASV_100	Bacteria	Bacteroidota	Bacteroidia	Cytophagales	Cytophagaceae	Sporocytophaga	Unclassified
ASV_127	Bacteria	Bacteroidota	Bacteroidia	Sphingobacteriales	Sphingobacteriaceae	Mucilaginibacter	Unclassified
ASV_408	Bacteria	Bacteroidota	Bacteroidia	Sphingobacteriales	FFCH9454	FFCH9454	metagenome
ASV_410	Bacteria	Bacteroidota	Bacteroidia	Sphingobacteriales	KD3-93	KD3-93	Unclassified

ASV_164	Bacteria	Chloroflexi	Ktedonobacteria	B12-WMSP1	B12-WMSP1	B12-WMSP1	uncultured_Chloroflexi
ASV_305	Bacteria	Chloroflexi	Ktedonobacteria	B12-WMSP1	B12-WMSP1	B12-WMSP1	uncultured_Chloroflexi
ASV_229	Bacteria	Gemmatimonadota	Gemmatimonadetes	Gemmatimonadales	Gemmatimonadaceae	Unclassified	Unclassified
ASV_309	Bacteria	Patescibacteria	Saccharimonadia	Saccharimonadales	WWH38	WWH38	uncultured_bacterium
ASV_348	Bacteria	Patescibacteria	Parcubacteria	Candidatus_Jorgensenbacteria	Candidatus_Jorgensenbacteria	Candidatus_Jorgensenbacteria	Candidatus_Adlerbacteria
ASV_671	Bacteria	Patescibacteria	Saccharimonadia	Saccharimonadales	LWQ8	LWQ8	uncultured_bacterium
ASV_32	Bacteria	Proteobacteria	Gammaproteobacteria	Pseudomonadales	Pseudomonadaceae	Pseudomonas	Unclassified
ASV_111	Bacteria	Proteobacteria	Gammaproteobacteria	Pseudomonadales	Moraxellaceae	Cavicella	uncultured_bacterium
ASV_403	Bacteria	Proteobacteria	Gammaproteobacteria	Burkholderiales	Comamonadaceae	Pelomonas	Burkholderia_sp.
